# Analytical Techniques for the Characterization of Bioactive Coatings for Orthopaedic Implants

**DOI:** 10.3390/biomedicines9121936

**Published:** 2021-12-17

**Authors:** Katja Andrina Kravanja, Matjaž Finšgar

**Affiliations:** Faculty of Chemistry and Chemical Engineering, University of Maribor, Smetanova 17, 2000 Maribor, Slovenia; katja.kravanja1@um.si

**Keywords:** bioactive coatings, orthopedic implants, characterization techniques, controlled drug release

## Abstract

The development of bioactive coatings for orthopedic implants has been of great interest in recent years in order to achieve both early- and long-term osseointegration. Numerous bioactive materials have been investigated for this purpose, along with loading coatings with therapeutic agents (active compounds) that are released into the surrounding media in a controlled manner after surgery. This review initially focuses on the importance and usefulness of characterization techniques for bioactive coatings, allowing the detailed evaluation of coating properties and further improvements. Various advanced analytical techniques that have been used to characterize the structure, interactions, and morphology of the designed bioactive coatings are comprehensively described by means of time-of-flight secondary ion mass spectrometry (ToF-SIMS), X-ray photoelectron spectroscopy (XPS), Fourier transform infrared spectroscopy (FTIR), atomic force microscopy (AFM), scanning electron microscopy (SEM), transmission electron microscopy (TEM), 3D tomography, quartz crystal microbalance (QCM), coating adhesion, and contact angle (CA) measurements. Secondly, the design of controlled-release systems, the determination of drug release kinetics, and recent advances in drug release from bioactive coatings are addressed as the evaluation thereof is crucial for improving the synthesis parameters in designing optimal bioactive coatings.

## 1. Introduction

The demand for orthopedic surgery is increasing with the aging of the population. Joint replacement due to osteoarthritis is one of the most commonly performed orthopedic procedures. Therefore, the development of suitable implants is crucial, especially due to frequent revision surgeries, which occur mainly due to aseptic loosening [1]. To ensure the adequate durability of an implant, the criteria for material selection must be met in terms of the mechanical properties (the modulus of elasticity, strength, ductility, etc.), surface roughness, corrosion resistance, and biocompatibility. Generally, implant materials consist of various polymers, ceramics, and metals (e.g., pure titanium, titanium alloys, stainless steel, and cobalt-chromium alloys) that possess adequate mechanical and corrosion-resistant properties, but which often do not exhibit the biological response that is key to successful osseointegration [1,2,3].

Osseointegration is defined as the direct structural and functional connection between the living bone and the surface of the load-bearing implant, which results from numerous cellular and extracellular biological processes [4]. Cell adhesion can be strongly influenced by the topographic features of the implant (hydrophilicity, roughness) [5,6,7]. Drug-eluting porous implants and biodegradable implants that degrade over time and are replaced by healthy body tissue have also attracted considerable interest [8,9]. However, another relatively simple approach leading to successful osseointegration and long-term stability can be achieved by coating implants with bioactive coatings that eliminate implant biological inertness and promote tissue–implant bonding at the interface [10,11,12,13,14,15].

Ideal bioactive coatings have the following properties: osteoinductivity, osteoconductivity [4], biocompatibility, an anti-inflammatory response [16], antimicrobial activity [17,18], corrosion mitigation, as well as suitable mechanical properties [19]. They can be composed of bioactive materials; various polymers [20], hydroxyapatite (HA) [21], calcium phosphate (CaP), titania nanotubes (TNTs) [22], carbon nanomaterials [23], etc.; they can also contain active substances that are released from their structure into the local environment [20]. Accordingly, the incorporation of anti-inflammatory drugs, such as nonsteroidal anti-inflammatory drugs (NSAIDs), can greatly reduce postoperative inflammation, as well as improving the osteointegration of the implant by promoting osteoblast proliferation [24]. Furthermore, the inclusion of antibiotics in implant coatings is recommended, as improper surgery and postoperative contamination from nearby tissues or hematogenous sources often lead to infections [25]. The addition of growth factors and osteoclast inhibitors has a positive effect on the quality of newly formed bone tissue [26,27,28,29,30], whereas the addition of anticoagulants may reduce the risk of clot formation and improve the blood compatibility of the biomaterials without affecting cell proliferation [31]. A number of factors influence the kinetics of the drug release, with the selection of an appropriate coating material and coating deposition technique (e.g., 3D printing, electrospinning [32], electrophoretic deposition [30], dip coating [33], drop casting [34], sol-gel deposition [35], biomimetic deposition [36], layer-by-layer deposition (LbL) [15], anodization [37], etc.) being particularly important, as they dictate the final shape of the drug delivery system, thus allowing a controlled and prolonged release for up to several months [38,39,40,41,42]. The novelty of a bioactive coating can only be appreciated by characterizing it, particularly under the chosen synthesis conditions.

In this regard, the use of time-of-flight secondary ion mass spectrometry (ToF-SIMS), X-ray photoelectron spectroscopy (XPS), and Fourier transform infrared spectroscopy (FTIR) is presented below, as these methods can characterize bioactive coatings and provide insight into their chemical composition [43,44,45,46,47,48]. The employment of atomic force microscopy (AFM), scanning electron microscopy (SEM), transmission electron microscopy (TEM), and 3D-tomography is also reviewed as they can provide visual information regarding the topography and morphology of such coatings [49,50,51]. Moreover, the use of a quartz crystal microbalance (QCM) is presented as this technique provides information regarding the material adsorption/desorption [52]. In addition, the use of adhesion tests is presented, which are performed to determine whether a coating attaches sufficiently to the substrate, and lastly, contact angle (CA) measurements are described as they play an important role in determining the hydrophobic or hydrophilic character of the material surface [53,54].

The aim of this review is to comprehensively discuss and critically evaluate recent advances in two segments of the characterization of bioactive coatings: (1) characterization techniques used to obtain interaction and morphology data, and (2) in vitro drug release testing, which is essential for optimizing controlled release formulations to achieve the desired release kinetics.

## 2. Interaction and Morphology Studies

Understanding the interactions and morphology of products obtained for specific applications is essential in order to facilitate the future development of improved, higher-quality products by varying the processing conditions [55,56]. Coupling techniques for the determination of chemical composition with techniques for the determination of morphology, topography, internal structure, and other surface-specific features enables a comprehensive analysis of bioactive coatings. Furthermore, such a characterization provides insight into the relationships of the obtained physiochemical properties with drug delivery and biological activity results.

### 2.1. Techniques for the Determination of the Chemical Composition of Coatings

#### 2.1.1. ToF-SIMS Analyses

ToF-SIMS is an analytical technique for analyzing surfaces up to a few nanometers in depth. The technique is based on measuring the intensity of selected ions as a function of the mass-to-charge (*m*/*z*) ratio of the ions. The ions are sputtered from the surface of a solid material that is stable under an ultra-high vacuum. This material is bombarded with high-energy primary ions. Hydrogen can also be analyzed using SIMS, which is frequently a disadvantage of other surface analytical techniques. Following XPS and Auger electron spectroscopy, SIMS is probably the third most widely used technique for surface analysis. The surface is usually bombarded (primary beam) with Bi^+^ ions (Bi^2+^ or Bi^3+^ ions are also used, depending on the type of sample). The bombardment with a primary ion beam slowly sputters the surface, producing mainly neutral atoms and a small proportion of positive (and negative) secondary ions—these are then separated in a mass analyzer to measure the mass spectrum. Thus, the mass spectrum can be the mass spectrum of the positive ions or the mass spectrum of the negative ions. Nowadays, SIMS devices using a time-of-flight (ToF) analyzer with a mass resolution of 30,000 are already available. Furthermore, a substantially higher mass resolution can be achieved in combination with an Orbitrap mass analyzer. The exceptional strength of the SIMS technique is the acquisition of 2D and 3D images. Typically, the image is obtained on an area of 500 by 500 microns at a resolution of 512 by 512 pixels. In this case, each pixel image represents an area of less than 1 micron [57,58,59].

**Figure 1:**
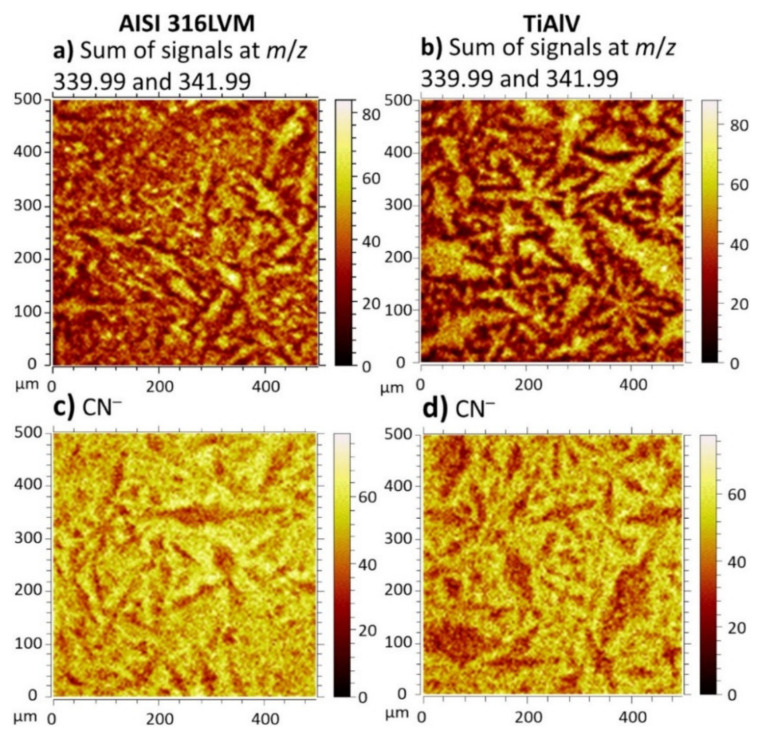
ToF-SIMS mapping of the coating on AISI 316LVM (**a**,**c**) and TiAlV (**b**,**d**) performed by summarizing positive ions at *m*/*z* 339.99 and 341.99 (**a**,**b**) and negative ion sat *m*/*z* 26.00 for CN^−^ (**c**,**d**) [57]. Reprinted from *Progress in Organic Coatings*, 158, Finšgar et al., *The development and characterization of bioactive coatings for local drug delivery in orthopedic applications*, Copyright (2021), with permission from Elsevier.

The ToF-SIMS technique is very useful in the characterization of bioactive coatings as it provides insight into the surface composition and elemental/molecular distribution. The use of this technique was demonstrated for a bioactive coating composed of polymethacrylates and sodium deoxycholate that was LbL-loaded with NSAID diclofenac (DCF) and applied on AISI 316LVM stainless steel and Ti6Al4V. Both polymer- and DCF-specific molecular signals or their fragments were identified in the negative ion and positive ion ToF-SIMS spectra. The DCF distribution in the coating was in the form of needle-like crystals, which was determined by 2D imaging, as depicted in Figure 1 [57].

Moreover, Michiardi et al. [60] reported a linear correlation of the ToF-SIMS intensities at *m*/*z* corresponding to S^−^, SO^−^, and TiO_3_H_2_^−^ (which were specific to the components in the coatings) and the XPS atomic percentage concentrations of S and Ti, suggesting the use of the ToF-SIMS technique for quantification purposes. In general, however, due to the complex matrix effects, the latter is unusual for the ToF-SIMS technique. On the other hand, ToF-SIMS signals are more selective for species in bioactive coatings compared with XPS signals. Furthermore, ToF-SIMS enables 3D imaging and the determination of the spatial distribution of species inside bioactive coatings. The latter is obtained by means of depth profiling using a secondary ion beam for sputtering (e.g., gas cluster ion beam (GCIB) and possibly also C_60_^+^/C_60_^++^ for organic materials, and Ar^+^, O_2_^+^, Cs^+^, Ga^+^ sputter beams for inorganic materials) associated with intermediate 2D imaging in pulsed mode. With the recent development of a GCIB sputtering source, the depth profiling of organic materials without significant changes in the material chemistry during sputtering provides new opportunities for material characterization, making this evolving technique very useful for the analysis of bioactive coatings in the future [58,59].

An example of ToF-SIMS 3D depth profiling has been presented for RF magnetron-sputtered CaP coatings of different thicknesses on Mg alloy implants to confirm the successful deposition and 3D distribution of species in coatings with a thickness of 70 nm and 210 nm [61]. Moreover, depth profiling, which indicates the different intensities of phase-associated ions over time, has been shown to be useful in detecting different phase transitions, starting with the surface of plasma-deposited coatings embedded with silver nanoparticles, followed by the implant material. Depth profiling additionally allowed a detailed analysis of aging in saline solution (0.15 M NaCl) after 60 days. A decreased thickness of the coating and the release of silver during aging in aqueous solution were reported. Furthermore, an increased ^35^Cl^−^ signal was detected near the surface, indicating that the plasma coating is rich in chlorine [62].

#### 2.1.2. XPS Analysis

XPS is a surface analysis technique, commonly used to characterize bioactive coatings on surgical implants, as it provides qualitative information on the elemental composition and valence state of the elements present in the bioactive coating [46]. In addition, it offers the possibility of quantitative analysis, which is not possible with the ToF-SIMS technique, for example. Most of the studies are performed with an Al K_α_ excitation source, and the scale is corrected at a binding energy (BE) of 284.8 eV (some employ 285.0 eV) based on the C-C/C-H signal in the C 1s spectrum of the analyzed species containing such features in their structure or due to the presence of adventitious carbonaceous species on the surface. XPS is a surface-sensitive method that provides information for a sample thickness of about 1–10 nm (depending on the angle of analysis and the inelastic mean free path of electrons traveling from the analyte through the surface layer). It is considered the most widely used technique for surface analysis in general and is usually used to perform survey and HR spectral measurements, XPS parallel imaging, GCIB, or monoatomic argon ion sputtering in association with XPS spectra measurements. After obtaining an XPS image, various chemometric techniques can be used to determine the distribution of phases on the surface [63].

In addition to ToF-SIMS, XPS depth profiling can be used to evaluate changes in the elemental composition, oxidation state, and formation of specific bonds with sputtering time. By measuring the depth of the crater created during sputtering (e.g., using AFM or 3D-profilomery), the sputtering depth can be determined. In this context, a low-damage GCIB ion source is often used, as the combination of XPS and GCIB has greatly expanded the range of materials that can be analyzed in depth by allowing the progressive removal of the surface without excessive chemical damage [64,65]. However, to the best of the authors’ knowledge, GCIB has been employed only once for bioactive coating analysis [66].

Using XPS, it is possible to assess the composition of bioactive coatings and thus the success rate of their deposition [61,67]. For example, XPS was used to investigate the AZ31 Mg alloy coated with calcium phosphate (CaP) of 70 nm and 210 nm thickness, which were prepared by means of RF magnetron sputtering. XPS high-resolution (HR) Mg 1s spectra for the uncoated AZ31 Mg alloy exhibited an intensive peak at a BE of 1304 eV for Mg and at a BE of 1307 eV for MgO. The presence of the CaP-coated alloy was confirmed by means of the corresponding Ca 2p and P 2p peaks [61]. XPS can also be used to evaluate the formation of an apatite layer after the immersion of the coating in simulated body fluids (SBFs). HR spectra were recorded for hybrid organic–inorganic TEOS–MTES (tetraethylorthosilicate–methyltriethoxysilane) sol-gel coatings applied on AISI 316L stainless steel implant material. Based on the Fe 2p, O 1s, Ca 2p, and P 2p HR spectrum measurements for the samples immersed in SBF, the in vitro bioactivity was confirmed by formation of apatite on the implants. In addition, XPS was used to determine the Ca/P, O/P, and O/Ca ratios for all samples after their immersion in SBF, confirming the formation of HA [68]. Using XPS, insights into the chemical interactions during the formation of the coating can be gained, which is crucial for understanding the final performance of the coating [69,70,71]. De Santis et al. [69] used XPS to analyze Ti implant samples with TNT-Ce_n_ (n = 1, 3, 6, 9, and 12, based on the number of depositions) coatings. It was reported that Ti was fully oxidized to Ti^4+^ due to the pure TNTs employed. Furthermore, it was observed that the Ce^3+^ ions from the precursor solution formed Ti-O-Ce bridges. The Ce^3+^/Ce^4+^ ratio was measured as it was found that this controls the enzyme-like behavior of the oxide. This ratio was found to be approx. 1:1, which is desirable and demonstrates the importance of both ions, since Ce^4+^ allows greater anti-inflammatory and osteogenic activity, whereas Ce^3+^ is able to bind phosphate species. In another study, XPS was used to determine the composition of rhBMP-2 immobilized on glycidyl methacrylate (GMA) prepared on a Ti substrate via initiated chemical vapor deposition (iCVD) [71]. XPS analysis of the bare Ti surface showed the most intense peaks for Ti 2p, C 1s, O 1s, and a less intense peak for N 1s. Ti 2p and N 1s disappeared with the deposition of the pGMA coating; however, the N 1s peak reappeared after the deposition of rhBMP-2 (a N at.% of 5.1% was reported). The strong chemical bonding of rhBMP-2 with pGMA was confirmed by determining the N/O ratio compared to rhBMP-2 physically adsorbed on the Ti substrate. The Ti-pGMA-BMP-2 sample exhibited a significantly higher N/O ratio, confirming the successful immobilization of rhBMP-2 on Ti-pGMA by an epoxide ring-opening reaction. Moreover, using XPS, the effect of the pretreatment of the bare implant surface can be evaluated prior to coating application. Hong et al. [72] identified the functional groups formed on the polyether ether ketone (PEEK) substrate after its treatment with sand blasting and acid etching. Subsequently, bioactive glass (BG) and chitosan (CH) were applied through dip coating to investigate the adhesion of the coating on the substrate. Using XPS, it was found that acid etching of the surface of the PEEK substrate contributed to the formation of the oxyl groups and consequently to the hydrophilicity of the surface and enhanced the spreading of the BG-CH coating solution.

#### 2.1.3. FTIR/ATR-FTIR

Fourier transform infrared spectroscopy (FTIR) is an affordable technique that can be used to characterize and identify various organic and inorganic materials. If certain species absorb the IR light of a molecule, there must be a net change in the dipole moment when the molecule vibrates or rotates. Only in this manner can the alternating electric field of light interact with the molecule and cause a change in the amplitude of one vibrational state. The vibrations occur at different wavenumbers (or frequencies) and are specific to certain bonds, thus serving as a characteristic tool for identifying particular species. The typical FTIR spectrum shows transmittance or absorbance (%) on the *y*-axis and wavenumbers (cm^−1^) on the *x*-axis, and consists of a series of peaks, each representing specific chemical bonds. Since the location of the peak (usually in the wavenumber range from 4000 to 600 cm^−1^) represents the specific chemical feature, whereas the height of the peak depends on the concentration of that species, both qualitative and quantitative analyses can be performed. It has to be emphasized that even though quantitative information can be obtained using FTIR, the latter is not used frequently [73,74,75,76,77,78].

**Figure 2:**
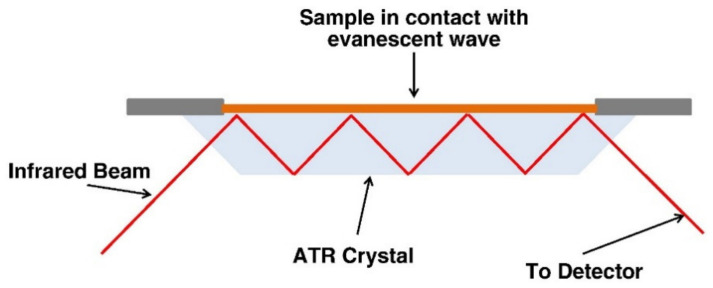
IR radiation path in the ATR-FTIR system [81]. Reprinted from Biochimica et Biophysica Acta (BBA)—Biomembranes, 1828, Yechiel Shai, *ATR-FTIR studies in pore forming and membrane induced fusion peptides*, 2306–2313, Copyright (2013), with permission from Elsevier.

Traditionally, IR spectroscopy has been used to characterize materials by transmitting IR radiation through the sample. In recent years, attenuated total reflectance Fourier transform infrared spectroscopy (ATR-FTIR) has attracted much attention due to the ease of sample preparation, its improved S/N ratio, and the ability to collect measurements in the presence of water [79,80]. The sample is brought into close contact with a transparent crystalline material (ATR crystal) with a high refractive index. As shown in Figure 2, the IR radiation is internally reflected several times before reaching the detector. The internal reflectance results in penetrating radiation, known as an evanescent wave, which extends beyond the crystal, penetrates a few micrometers into the sample, and is attenuated in the regions of the IR spectrum where the sample absorbs energy. The attenuated energy of the evanescent wave is passed back to the IR beam, and finally the attenuation of the beam, called the attenuated total reflectance, is measured by the detector [77,79].

In the characterization of bioactive coatings, FTIR/ATR-FTIR can be used to provide information regarding the chemical composition of such coatings and the bonding that occurs during deposition, whereas in multilayer coatings the number of bilayers can be assessed according to the increased absorbance intensity [67,69,82,83,84]. Moreover, this technique can provide information regarding bioactivity. Using ATR-FTIR, the bioactivity of prepared coatings was confirmed by signals related to PO_4_^3^^−^, indicating apatite, in a study by De Santis et al. [69] after ceria was combined with TNTs and soaked in SBF at 37 °C for 8 days. The technique can also offer information on whether the drug was successfully incorporated into the bioactive coatings and whether it was physically or chemically bound to the coatings [32,34,85,86,87,88,89,90]. In a study by Kiran et al. [87], coatings of poly(ε-caprolactone) (PCL) nanofibers loaded with HA and rifampicin were electrospun on a Ti substrate. FTIR analysis revealed that the drug remained intact in the system and that little to no dihydroxylation occurred in the case of HA, as indicated by the OH^−^ related peaks in the spectra. Moreover, using FTIR, the chemical composition of the coatings can be compared before and after immersion in SBF during in vitro drug release testing, focusing on the degradation of the coating and the completeness of the drug release [39,91]. However, the major drawback of this technique is its inability to distinguish between functional groups when the peaks in the spectra overlap [82,92]. Table 1 shows the functional groups and corresponding wavenumbers (cm^−1^) for some typical bioactive coating materials and the drugs contained in the coating materials.

### 2.2. Techniques for the Determination of Morphology, Topography, and Internal Structure

#### 2.2.1. AFM (At Nanoscale)/Profilometry (Larger Area)

By means of the AFM technique, completely nonconducting substances can be studied, which is not possible with the STM technique. Compared to scanning tunnelling microscopy (STM), with AFM forces are measured, rather than current. It works on the principle of measuring forces between the tip and the sample. The forces between the tip and the sample can be short-range or long-range. AFM can achieve a magnification of up to 1,000,000 times, and the distance can be measured in the vertical direction. The advantage of AFM over SEM is the true 3D resolution of the profile and the atomic resolution capabilities. AFM can be performed under atmospheric conditions, in a vacuum, and in liquids. The AFM instrument consists of a cantilever (often made of silicon nitride or silicon or tungsten) that has a sharp tip with a radius of curvature of a few nm (i.e., a very sharp tip). When the tip approaches a certain surface (e.g., in AFM contact mode), atomic forces act between them, causing the cantilever to bend, which is measured via the reflection of the laser beam. The forces that affect AFM measurement vary: van der Waals, electrostatic, magnetic, capillary, ionic, and repulsive forces. The AFM technique is often used in biomedical applications [98].

When applying bioactive coatings to metal substrates, AFM provides an opportunity to characterize the topography and measure the roughness parameters, both of which have an important influence on the osseointegration and interfacial stability of the implants [68,99,100]. Table 2 summarizes a number of studies focusing on the preparation of bioactive coatings on metallic substrates that have been characterized using AFM. The coating deposition technique and implant and coating materials are given, along with the AFM measurement setup and the results obtained.

#### 2.2.2. SEM/EDX (SAXS)

SEM is used to visually characterize the morphology of organic and inorganic materials on a nanometer to micrometer scale using a narrow electron beam that rasters a scan pattern on the sample’s surface. HR grayscale images with up to 300,000-times (1,000,000-times has also been reported) magnification can be obtained through the detection of the secondary electrons and the backscattered electrons from a sample surface coated with gold or palladium ions [103,104,105].

This technique, therefore, allows a comparison of the morphological characteristics of bare metal implants with those of coated implants [34,40,87,106], as well as a comparison of prepared bioactive coatings with different compositions [107]. It can additionally help to determine the morphological changes of coatings at different time intervals in SBF after in vitro drug release tests [108]. In addition to the morphology and microstructure analysis of the coating surface [88,92,109] or cross sections [67,83,91], the thickness of the coatings and the adhesion of osteoblasts on coated implants can be investigated [67,110,111]. For example, Eawsakul et al. [110] used SEM to measure the thickness of PLGA/BMP-2 coating on Ti and to study osteoblast adhesion. The results showed the promotion of cell growth with an increasing amount of BMP-2 entrapped on the implants. Moreover, SEM enables the determination of the diameters of pores, nanotubes, or other active ingredient carriers, as well as the calculation of particle size and distribution when loading coatings with active ingredients [39,86,94,112]. The nanoparticle size distribution is an important parameter in the design of the coating system, since the release and degradation of the active compound in SBF depends on the availability of the surface reactive area in contact with the surrounding media. Aydemir et al. [39] used SEM to determine the particle size and distribution of an electrophoretically deposited chitosan-gelatin coating with silica-gentamicin nanoparticles on surgical grade stainless steel. The shape and distribution of the particles were analyzed by means of digital image processing using an automatic algorithm described in a study by Meng et al. [113]. The analysis of nanofiber FE-SEM (FE—field emission) micrographs with ImageJ software additionally allows the calculation of the pore surface area and porosity percentage [112].

Although SEM provides visual information, it is not a quantitative technique on its own. However, when combined with other techniques, valuable data can be obtained [114]. Energy dispersive X-ray spectroscopy (EDXS, frequently abbreviated only EDX or EDS) is performed in association with SEM and provides quantitative data (based on the intensity of the emitted X-rays). By measuring the intensity of the X-rays, it is possible to produce chemical maps, as well as to obtain quantitative data on the elemental composition of the bare substrate and coatings as an atomic percentage (at. %) [61,83,105,115]. For example, Acheson at al. [61] used SEM/EDXS to determine the elemental composition and to calculate the Ca/P ratio for CaP coatings. On the other hand, Ballarre et al. [68] used small-angle X-ray scattering (SAXS) to analyze TEOS-MTES sol-gel coatings on AISI 316L stainless steel. They were able to determine the values of mean mineral thickness and the degree of orientation of mineral particles in old cortical bone and the newly formed bone after implantation. SAXS analyzes the elastic scattering behavior of X-rays as they pass through the sample and measures their scattering at small angles (0.1°–10°) [116]. Quantification is performed based on nanoscale density differences and information can be obtained for systematic structural analysis, such as particle shape, thickness, orientation, and arrangement in composite coatings [117,118].

#### 2.2.3. TEM

TEM also employs an electron beam to obtain high magnification images (like SEM). However, it differs from SEM in regard to (1) its ability to obtain higher resolution, (2) its main principle, as the electron beam (100–1000 keV) is transmitted through a sample, losing energy as the electrons pass through electron-dense regions, which is detected with the help of a fluorescent screen, and (3) its difficulty obtaining 3D information, as the specimen needs to be viewed and scanned from many directions for this purpose [119,120,121].

Using this technique, it is possible to study the substrate surface and structure along with the morphology of the coatings and the loaded drugs [86,122]. Enrofloxacin, which was functionalized on TNTs by -SH and -NH_2_ surface-grafted groups, was analyzed using TEM. The gaps between the TNTs were found to be filled with the drug, resulting in an indistinct shape of the previously tubular structure [85]. Determination of the size distribution, surface roughness [39], shape, and particle size [99,123], as well as observation of the cross-section morphology, are also possible [124,125]. Furthermore, similarly to SEM, TEM can be coupled with EDXS for the elemental quantification of substrates and coatings [126,127]. For example, an HA coating on a Ti substrate was prepared through a combination of microarc oxidation (MAO) and microwave hydrothermal treatment (MH). The microstructure of the obtained coatings was investigated by means of TEM, the Fast Fourier Transform (FTT) technique was utilized to study the crystal structures, high-angle annular dark field (HAADF) images were obtained to inspect the Z-contrast, and the elemental mapping distribution and line scanning were determined by means of EDXS in combination with the TEM system. After MAO treatment, porous coatings were observed, which were mainly composed of Ca, P, Si, Ti, and O. A large number of HA crystals were visible on the surface after 10 min. of MH treatment; however, when MH treatment was extended to 60 min., the crystals dissolved and Na_0.23_TiO_2_ was produced after TiO_2_ reaction with OH^−^ ions [126].

#### 2.2.4. 3D-Tomography

X-ray computed tomography (CT) provides tomographic (cross-sectional) images by scanning specimens with X-rays and processing them on a computer using reconstruction algorithms [128]. Although the application of CT is usually aimed at medical examinations, micro-CT and nano-CT technologies have been used for the characterization of various biomedical materials.

**Figure 3:**
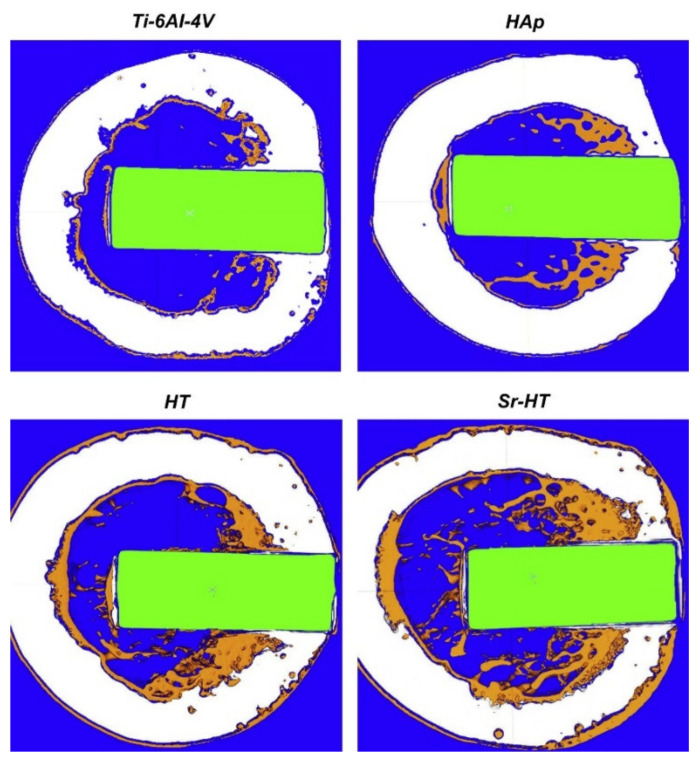
Micro-CT images of the transverse sections with a radius of 1 mm from implant surface of a canine femur 12 weeks after the implantation of bare Ti alloy, HA-, HT-, and Sr-HT-coated implants [138]. Reprinted from Biomaterials, 34, Zhang et al., *The synergistic effect of hierarchical micro/nano-topography and bioactive ions for enhanced osseointegration*, 3184-3195, Copyright (2013), with permission from Elsevier.

A typical micro-CT device consists of a micro-focus X-ray source, a rotating stage, a flat-panel detector, and a computer. The X-rays penetrate the rotating sample and 2D projections of different angular positions can be obtained. Subsequently, 3D micro-CT image reconstruction is possible by means of processing software, for example, by using the Feldkamp–Davis–Kress (FDK) algorithm [129,130]. This has been proven useful in gaining insight into the morphology of bioactive coatings, usually focusing on the determination of porosity [131,132]. The corrosion rate of implant materials can also be assessed. In a study in which an AZ31 alloy was coated with CaP coatings of varying thickness, volumetric analysis of the uncoated and coated AZ31 samples was performed using micro-CT after they had been immersed in SBF for 14 days. The observed volume loss was greater in the case of bare samples, indicating that the CaP coatings successfully serve as a protective barrier that reduces corrosion in AZ31 alloys [61]. Nevertheless, the technique has been primarily used to quantify bone growth by segregating bone tissue from the implant after in vivo implantation and to calculate the percentage of regenerated bone volume/total volume (BV/TV) [133,134,135,136]. Qiao et al. [137] fabricated 3D-printed Ti6Al4V coated with platelet-rich plasma (PRP) and implanted it into rabbit models. Months after the implantation, micro-CT images confirmed an increase in the amount of tissue present. In another study, strontium-substituted hardystonite (Sr-HT) ceramic coatings were applied to a Ti alloy. The new bone formation was evaluated for the obtained coated implant, along with the HA-coated and uncoated Ti alloy. Figure 3 shows the micro-CT images 12 weeks after implantation, with the highest amount of newly formed tissue clearly seen in the case of the Sr-HT ceramic coatings [138].

Nano-CT differs from micro-CT in the utilization of a nano-focus X-ray source. It performs scans with a nanometer-scale resolution and therefore yields images of greater detail. It is also evident that nano-CT provides better 3D spatial visualization than conventional micro-CT, SEM, or AFM methods [139]. It offers insights into the morphology (e.g., crystallinity and porosity) of bioactive materials prepared under different conditions [140,141,142] and can be used to evaluate bone growth after implantation, similarly to micro-CT examinations. In an in vivo study carried out by Cuijpers et al. [143], the authors compared micro- and nano-CT techniques for the assessment of newly formed bone tissue after the implantation of Ti-coated PMMA implants. It was discovered that although nano-CT provided significantly higher histomorphological details of the implant and surrounding tissues, the quantification of the newly formed tissues (such as bone area, bone-implant contact, and bone volume percentage) was more representative in the case of micro-CT due to the larger samples that can be measured with this technique (in the centimeter range). Therefore, both of these techniques have certain advantages and should be used in a complementary manner.

### 2.3. QCM

Quartz crystal microbalance (QCM) is a technique that measures the change in frequency that is proportional (according to the Sauerbrey equation) to the change in mass in the nanogram-to-microgram range, and can be used to determine the amount of material adsorbed on a substrate surface in real time. A decrease in the vibration frequency indicates an increase in the thickness of the adsorbed material. The device consists of a piezoelectric quartz crystal located between the top and bottom electrodes, and starts to vibrate upon the application of potential, which causes the crystal to vibrate at a resonance frequency [144,145,146,147].

QCM is often used to evaluate the adsorption or desorption of coatings on a substrate. For example, it was recently reported that the protein amelogenin adsorbs better on a Ti surface when the Ti is nano-modified to form titania nanosheets [148].

Different QCM variations are also available. In particular, quartz crystal microbalances with dissipation monitoring (QCM-D) have gained popularity in recent years as this approach allows the measurement of adsorbed mass and viscoelasticity. Monitoring the change in resonance frequency (Δ*f*) and the dissipation factor (ΔD) for several overtones is performed by recording the oscillation decay curve after the power is turned off [149]. QCM-D has been used to obtain information regarding the build-up of novel organic-inorganic LbL coatings based on BG, CHI, and hyaluronic acid modified with catechol groups. The viscoelastic properties were evaluated based on the Voigt model, whereas the thickness was calculated by fitting the Δ*f* and ΔD by implementing the Simplex algorithm [150]. In addition, QCM-D has been utilized to evaluate the adsorption and stability of novel bioactive coatings of CHI and anionic surfactant 77KS, which served as a drug delivery system for amoxicillin and were applied to polydimethylsiloxane, i.e., Δ*f* and ΔD were observed at the 3rd overtone, the first indicating the mass adsorption of the different combinations of the prepared coatings, and the second the viscoelasticity. Using QCM-D, the successful adsorption of prepared bioactive coatings with or without amoxicillin on the substrate was observed due to the physical and hydrophobic interactions. In contrast, the influence of the UV/ozone activation of the substrate and the presence of NaCl were found to be ineffective [151].

### 2.4. Coating Adhesion Measurements

The American Society for Testing and Materials (ASTM) defines adhesion as a condition in which one surface is attached to another as a result of interfacial bonding [152]. Methods for determining adhesion can be divided into three categories, namely, nucleation, mechanical, and miscellaneous methods. Nucleation methods are suitable for the determination of basic or atomic adhesion, whereas mechanical and miscellaneous methods are more suitable for the determination of practical adhesion. Adhesion measurements can be displayed as the force per unit area or as the work/energy required to separate the coating from the substrate. The work of adhesion is given by Equation (1):

(1) $$W_{a}=\gamma_{A}+\gamma_{B}-\gamma_{\mathrm{AB}}$$

where $W_{a}$ is the work of adhesion, $\gamma_{A}$ is the specific surface energy of the coating, $\gamma_{B}$ is the specific surface energy of the substrate, and $\gamma_{\mathrm{AB}}$ is the specific surface energy of the phase boundary between the coating and the substrate. If the work of adhesion is positive, the coating is well attached to the substrate, and the adhesion is sufficient, and vice versa if the work of adhesion is negative [53]. Various qualitative and quantitative methods are available to measure the practical adhesion [153]. It has to be pointed out that the results are comparable if the measurements are carried out with the same method and under the same conditions.

Some examples of qualitative adhesion tests, which are usually based on subjective judgement, are the knife test, bend test, and tape test. The knife adhesion test is described by the ASTM D6677 standard and is used to determine the adhesion between thick layers of soft organic coatings applied to solid substrates. The test is performed using a knife to make two incisions in the coating layer in the shape of a cross at a 30° to 45° angle. The tip of the knife is then inserted into the cross section in the coating in an attempt to remove the coating from the substrate. Adhesion is subjectively evaluated by the force required to remove the coating from the substrate, ranging from 0 to 10 using the criteria referred to in the given table [154,155]. The bend test is used for a variety of coatings to determine overall flexibility and adhesion and is based on bending the sample under shear stress. The test is performed with a round pin of which the diameter is at least four times as thick as the sample. The pin exerts a force on the sample to bend it until the coating is damaged [153]. The tape test is described by ASTM D3359 [156] and is most commonly used for thin organic and polymeric coatings. A sharp blade is used to cut a specific pattern (X-cut or cross-hatch cut) into the coating, followed by a pressure-sensitive adhesive tape with well-defined adhesive properties that is adhered to the coating. The adhesive tape is then peeled off of a sample and observed to see if it contains any coating residue [153,157]. Chen et al. [158] used a cross-hatch tape test to qualitatively determine the adhesion between a 316L stainless steel substrate and an alginate/Bioglass^®^ composite coating obtained via electrophoretic deposition (EPD) with direct current (DC) or alternating current (AC). It was found that the maximum value of the peeled area was not greater than 15%. The number of EPD cycles decreased the adhesion strength when using DC EPD and remained unchanged when using AC EPD.

Quantitative tests, on the other hand, offer the ability to measure adhesion accurately and are therefore appropriate for more detailed experimental work. Some examples of quantitative tests are the scratch test, the pull-off test, the four-point bend test, and the peel test. The scratch test is described by the ASTM D2197 standard [159]. Its application is limited to measuring the adhesion of thin films on smooth and flat surfaces. The test is based on the horizontal movement of the sample under a weighted stylus tip to produce a scratch on the coating surface. The test can be performed with a constant load or with a linear increase in the load until the coating is removed from the substrate. The result obtained is the critical value of the load that occurs when the coating separates from the substrate. It can be determined by observing the scratch under a microscope, by measuring the coefficient of friction, or by analyzing the measured acoustic emission [160,161,162]. Wu et al. [163] applied sphene and HA coatings on Ti6Al4V disks and performed a scratch test to quantify the adhesion using a constant load and optical microscopy. The sphene coatings maintained their integrity and exhibited only minor scratches up to a load of 80 g-force, compared to the HA coatings, which failed at a load of 25 g-force, and thus exhibited better adhesion properties. Booth et al. [164], on the other hand, used the progressive load scratch test to evaluate the adhesion of their multilayered coatings consisting of alternating nanocrystalline (NCD) and microcrystalline (MDC) diamond to a Ti6Al4V substrate. The load rate was 2 N/s up to 60 N, whereas the scratch tracks were examined using a microscope. The results showed a high level of adhesion for all coatings; however, the highest critical load value was found for the single-layered NCD coatings. The pull-off test is described by ASTM D4541 [165] and is suitable for the majority of coatings. It consists of a dolly attached vertically to the coating and a screw that is gradually loaded by winding until the coating is pulled off the substrate. The results obtained are the critical tensile strength in psi or MPa [162,166]. Sharifnabi [167] employed the pull-off test to measure the adhesion between medical-grade AISI 316L stainless steel and a Mg-substituted fluorapatite coating obtained by means of the sol-gel dip coating technique. An automatic adhesion tester (PosiTest AT-A, DeFelsko) was employed to perform a pull-off test. A satisfactory adhesion strength was demonstrated as the maximum pressure of 4 MPa did not affect the coating. The four-point bend adhesion test provides quantitative results compared to the basic bend test, as it offers information on the deformation or load at which the coating is damaged. The method is based on the use of pins that are in contact with the sample at four points, which is placed horizontally between them. Two cylinders are placed at the bottom of the sample and two at the top, which are used to increase the surface tension by exploiting the load until damage to the coating occurs [166,168]. Hong et al. [72] used the standard pull-off and four-point bend tests to measure adhesion, with the PEEK implant substrate coated with chitosan/Bioglass^®^ composite coatings using the dip-coating method. The four-point bend test was additionally selected to simulate the stress conditions of spinal implants. The results for both tests were comparable and showed drastically improved adhesion by subjecting the substrate to sandblasting and acid etching treatment, compared to acid etching treatment alone. The peel test can be used for flexible coatings that can be bent at least 90° and have a coating thickness not exceeding 0.125 mm. The test is performed by attaching a handle to the coating and gradually applying a force to peel the coating from the substrate at either a 90° or 180° angle. The result is usually presented as the force required to peel the coating from the substrate per unit length of sample (N/25 mm or N/50 mm, depending on the width of the sample) [166,169,170]. Kurzweg and Heimann [171] demonstrated that the peel test is suitable for determining the adhesion between a Ti6Al4V substrate coated with thin layers of titania and zirconia (bond coating), followed by a thick layer of atmospheric plasma-sprayed HA. The results showed that the adhesion strength was significantly improved with the intervening bonding layers (especially titania layers) compared to HA deposited directly on a Ti6Al4V substrate.

### 2.5. Contact Angle Measurements

**Figure 4:**
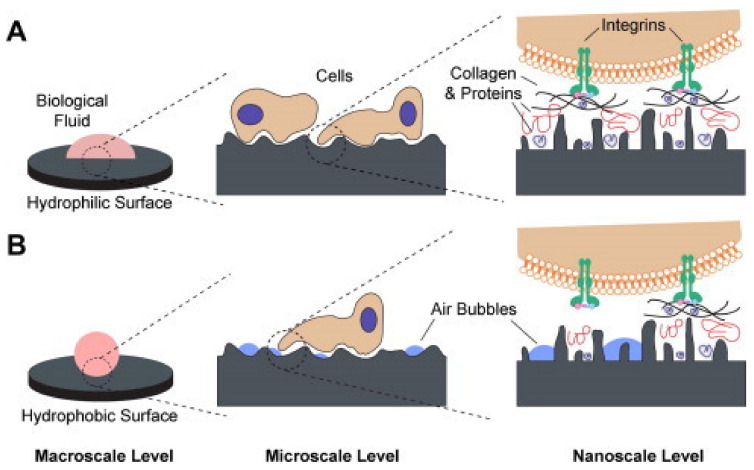
A schematic representation of potential biological interactions with (**A**) hydrophilic and (**B**) hydrophobic surfaces. (**A**) hydrophilic surfaces interact with biological fluids, allow adsorption of proteins to the material surface, and facilitate interaction with cell receptors. (**B**) hydrophobic surfaces generally contain hydrocarbon contamination and consequently entrap air bubbles that inhibit protein adsorption and cell receptor activation [184]. Reprinted from Acta Biomaterialia, 10, Gittens et al., *A review on the wettability of dental implant surfaces II: Biological and clinical aspects*, 2907-2918, Copyright (2014), with permission from Elsevier.

Contact angle measurements are performed to describe the ability of a solid substrate to repel a liquid and are considered to be a quantitative measurement of surface wettability [172,173]. Depending on the application, different methods can be used, such as the sessile drop method, the captive bubble method, the Wilhelmy plate method, etc. [174,175,176,177]. However, the main principle is to drop liquid (water) onto the surface and analyze the shape of the obtained drop, which changes as a result of various surface properties (surface roughness, surface energies, surface chemistry, and surface coatings) [173]. In the most common sessile drop measurements, the droplet is placed on a solid surface until it reaches an equilibrium of forces, meaning that the sum of the interfacial tensions in the plane of the surface is zero. This phenomenon is explained by Young’s equation (Equation (2)):

(2) $$\theta_{\mathrm{sv}}-\theta_{\mathrm{sl}}-\theta_{\mathrm{lv}}\cdot \cos\theta =0$$

where $\theta_{\mathrm{sv}}$ represents the solid-vapor surface tension, $\theta_{\mathrm{sl}}$ represents the solid-liquid surface tension, and $\theta_{\mathrm{lv}}$ represents the liquid-vapor surface tension [178]. The droplet is observed through a combination of precision optics, cameras, and sophisticated software, which allow easy and rapid determination of the contact angle [179]. The contact angle (θ) is geometrically defined as the angle formed by the liquid droplet at the three-phase boundary where liquid, gas, and solid intersect [173]. The method is well established for plain metal substrates, various films, and coatings [179,180]. It indicates the hydrophilicity and hydrophobicity of the material at issue, depending on the contact angle measured. Surfaces with a contact angle greater than 90° are considered hydrophobic (water repellent), whereas substrates with a contact angle less than 90° are defined as hydrophilic [179,180]. The hydrophilicity/hydrophobicity of coated implants has been reported to be closely related to the biological response, as it affects cell adhesion, cell proliferation, and protein adsorption (Figure 4) [180,181,182,183,184].

Values of 35°–80° have been found to be optimal for materials in biomedical applications, as cell adhesion and proliferation tend to be enhanced at high hydrophilicity, whereas values below 35° negatively affect protein attachment, which is known to cause thrombogenicity [181,185,186,187]. It has been shown that the coating of implants and their various surface modifications can improve hydrophilicity and thus accelerate osseointegration. For example, a variation in surface topography has been shown to influence surface wettability. In a study where CHI/BG coatings were deposited on TiAlV alloy by means of electrophoretic deposition, the effect of different surface treatment of TiAlV was observed through contact angle measurements. It was found that grit-blasted substrates were favorable as the wettability was significantly increased [181]. Furthermore, in another study, it was reported that pre-treatment of PEEK implants with an accelerated neutral atom beam (ANAB) resulted in nanometer-scale surface modifications and consequently increased surface hydrophilicity [188]. On the other hand, Cordero-Arias et al. [189] discovered higher hydrophilicity of composite chitosan/nano titania (nTiO_2_) on stainless steel by including higher concentrations of nTiO_2_. Therefore, it can be concluded that the hydrophilicity of implant surfaces can be improved by applying certain types of coatings. The hydrophilicity of metal-based implants has been improved by the application of TNT/nTiO_2_ [85], HA [190], BG [191], and various polymers [32,87,192]. Nevertheless, although contact angle measurements can provide information on the interactions between the surface and gases or liquids, other techniques should be employed to provide more detailed characterization of chemical properties, as described in the sections above [178].

## 3. Controlled Release

Over the years, several studies have focused on the preparation of localized drug release strategies for orthopedic implants by incorporating drugs into implant coatings [32,108,193,194]. Some of the advantages of a localized drug release over systemic drug delivery are the achievement of fewer side effects by avoiding systemic drug exposure, higher bioavailability as the drug is administered directly to the target tissue, a lower dosage needed to achieve the desired effect, and the ability to customize the release kinetics [38,195]. These benefits can be achieved through controlled release systems in which drugs or other active ingredients are incorporated into a carrier (implant coating) and are slowly and continuously released into the surrounding media over the desired period of time. The release rate is determined either by the microstructure of the carrier and the intermolecular interactions between the carrier and the drug, or by the environmental factors in which the carrier is located, such as the pH and temperature of the body fluids [196,197].

In general, controlled release systems can be classified into four classical mechanisms, namely, diffusion-controlled, chemically-controlled, solvent-activated, and magnetically-controlled mechanisms [196]. A diffusion-controlled mechanism is a mass transport mechanism that plays a major role in most controlled release systems and can be analyzed using Fick’s diffusion theory. Equation (3) represents Fick’s well-known First Law, which can be used to determine diffusion in a single direction, with the basic idea that a drug diffuses from a site of higher concentration to an adjacent site of lower concentration:

(3) $$J_{i}^{*}=-D_{\mathrm{ip}}\frac{dc_{i}\left(t,z\right)}{dz}$$

where $J_{i}^{*}$ represents the molar flux of the drug with respect to the molar average velocity of the system, $c_{i}$ represents the drug concentration, t represents time, z represents the thickness of the film, and D represents the diffusion coefficient or diffusivity [196,198]. The diffusion-controlled mechanism is further classified into a reservoir or a monolithic system, depending on the inner structure of the drug delivery system. The reservoir type refers to a drug being incorporated into a carrier in a core-shell arrangement, whereas the monolithic type refers to the homogeneous distribution of a drug in a carrier matrix [198]. A chemically controlled mechanism includes erodible systems, in which the drug is incorporated into a biodegradable carrier, with its release being affected by the degradation rate of the carrier material, or a pendant chain system, in which the drug is hydrolytically or enzymatically linked to a polymer and is released into the surrounding media after the linkage is broken [196]. The solvent-activated mechanism is determined by the permeation rate of the solvent and is further divided into osmosis and swelling. The osmotic delivery system consists of a carrier, acting as a semipermeable membrane, and an osmotic core containing an active agent. The solvent diffuses through the membrane to the core, generating the osmotic pressure and consequently regulates drug release into the surrounding media in zero-order kinetics [199,200]. The swelling mechanism, on the other hand, allows the release of the drug after the solvent diffuses into the carrier containing the dispersed drug, causing the carrier material to swell and release the drug in a controlled manner. Finally, magnetically-controlled systems contain magnetic beads in addition to drugs dispersed in a carrier. When exposed to an oscillating external magnetic field, the drug is released at a different (higher) rate than the usual diffusion-controlled rate [201].

**Figure 5:**
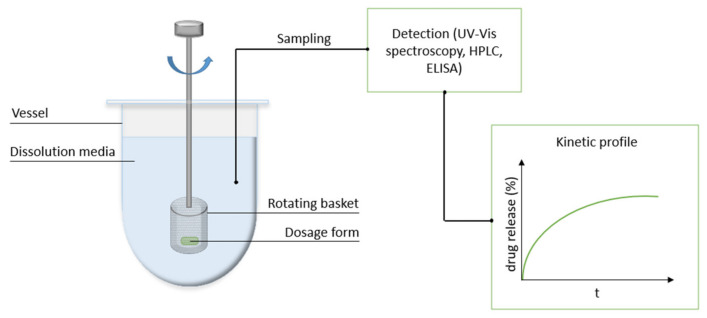
In vitro drug dissolution process with USP apparatus type I (rotating basket).

In vitro drug release tests have been shown to be useful in the early stages of controlled-release formulations because they allow the prediction of release behavior in vivo and the optimization of kinetics to achieve a controlled release. In addition, they reduce the experimental time, lower expenses, and are undoubtedly more ethical. Much research effort has been invested into the development of appropriate in vitro release testing methods and technologies to ensure simplicity, batch-to-batch reproducibility, and comparability with real in vivo body conditions [202,203]. In this regard, a variety of methods have been developed for in vitro drug release testing, generally involving the immersion of the carrier containing the selected drug in the prepared dissolution media, such as SBF, under well-defined conditions. This step is followed by sampling at different time intervals, supplying fresh dissolution media, the filtration/centrifugation of the sample taken [204], and drug detection in the sample using UV-Vis spectroscopy [108], enzyme-linked immunosorbent assay (ELISA) [40], or high-performance liquid chromatography (HPLC) [92]. Seven variants of United States Pharmacopeia (USP) apparatuses [202] (USP apparatus type I is shown on Figure 5) or other designs (e.g., Franz diffusion cells) are currently the most frequently used for in vitro drug release testing [57,203].

### 3.1. Release Models

Understanding the release kinetics is of the utmost importance in the development of controlled-release formulations, as it describes the in vitro and consequently the in vivo release processes and therefore enables the effective design and optimization of drug carriers [202].

The result of in vitro release tests are different kinetic profiles, which show the dependence of the concentration of the released drug and the corresponding release time. The release kinetics depends on the crystallinity, particle size, solubility, and amount of the drug [205]. A simple method to determine the kinetic model is to fit the experimental data (the concentration of the released drug as a function of time) to various linearized mathematical models, plot a graph, perform a linear regression of the plotted graph, and determine the correlation coefficient, R, and the square of the correlation coefficient, R^2^. The most appropriate kinetic model is the one with the R^2^ value closest to 1.00 [206]. Some of the most common kinetic models are presented in Table 3.

**Figure 6:**
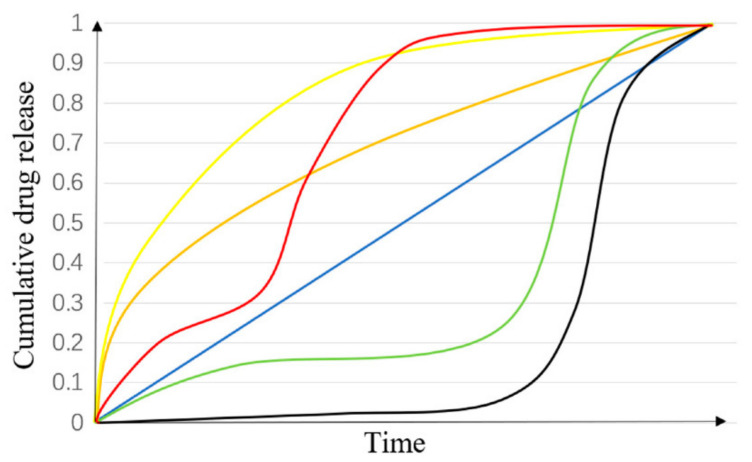
A triphasic drug release profile with a short second phase (red), a burst drug release profile (yellow), a burst release profile with zero-order (orange), a zero-order drug release profile (blue), a triphasic release profile (green), and a biphasic drug release profile (black). Reprinted with permission from [209].

It should be noted that although zero-order release is usually favored due to drug release at constant concentrations over time, it is not representative in drug release formulations. The most frequently obtained release profiles are triphasic, with phase I representing a burst release of the unencapsulated drug at the surface, with phase II being a slow release dominated by diffusion processes, and phase III a faster release of the drug as a result of nanofiber erosion, as shown in Figure 6 [38,209].

### 3.2. Drug Release from Bioactive Coatings

In the studies performed to date, a wide range of antihyperlipidemics [94], analgesic and anti-inflammatory drugs [91,108], antibiotics [210], bisphosphonates [30], selective estrogen receptor modulators [211], and growth factors [40] have been incorporated into medical implant coatings with the aim of preventing postoperative complications and inducing adequate acceptance and integration of the implant into the body. The drug release is strongly influenced by factors such as the type and physiochemical properties of the selected drug, the coating and the implant, the deposition technique, the focal tissue environment, and the method of incorporating the drug into the coatings, indicating different interactions between the drug and the coating material [38,212]. Hence, the drug release kinetics of the different novel coating-implant systems from recent studies are summarized in Table 4.

The major limitation of the currently manufactured bioactive coatings is their frequent inability to achieve the desired controlled and sustained release of drugs [212,216]. As shown in Table 4, the use of different coating materials and deposition techniques to modify their properties (e.g., composition, thickness, porosity, surface functionalization, etc.) can significantly affect the drug release kinetics in SBF. In most cases, the obtained release profiles indicate an initial fast release, followed by a slower release, and finally a plateau, all ranging from a few hours to a few months. It can be seen that some coating formulations prepared via electrospinning [87,108], electrophoretic deposition [41,114], dip coating [40], LbL [102], and anodization [214] may provide a drug release for more than a month.

## 4. Conclusions

Advances in materials science, cell biology, and pharmacology have facilitated the rapid development of novel bioactive coatings for orthopedic implants with the aim of promoting bone ingrowth into predominantly biologically inert implants. It should be emphasized that mechanically stable, biocompatible, antimicrobial, anticorrosive, osteoinductive, and osteoconductive coatings have already been produced through the careful selection of coating materials, drugs, and coating deposition techniques. In addition, the design of drug-eluting implant coatings for controlled drug delivery is also on the rise due to their important attributes as to the on-site prevention of postoperative complications, fewer side effects, and higher bioavailability at lower drug doses. Although the tremendous progress in relation to current bioactive coatings is evident, much research is still needed before they enter clinical practice.

In the development of multifunctional bioactive coatings, the optimization of their chemical interactions, structure, topography, surface wettability, mechanical properties, and drug release kinetics is fundamental and can only be assessed through proper characterization. On this basis, this review focused on the common characterization techniques used for interaction and morphology studies, as well as their basic principles and performance in the case of bioactive coatings. In addition, the concept of controlled drug delivery, the typical kinetics models, and recent advances in the field of drug-releasing bioactive coatings are addressed. Nowadays, the cellular response and the degree of biomineralization can be foreseen as a function of the surface properties of the coatings. Characterization of the topography and morphology of both the coatings and the cells adhering to them is performed by mean of AFM, SEM, TEM, and 3D tomography, whereas surface chemistry is determined using advanced techniques such as ToF-SIMS and XPS, which in combination with GCIB allow depth profiling without major sample damage, or by ATR-FTIR analysis. Although the aforementioned techniques allow the qualitative analysis of the obtained coatings, quantitative analysis is only possible with XPS, SEM/EDXS, and ATR-FTIR. In contrast, the advantages of ToF-SIMS are its low detection limit, its determination of the presence of isotopes, and the possibility of 2D and 3D imaging to show the distribution of the components in the samples. Measurements of coating adsorption or desorption are possible with the QCM technique. A wide range of versatile quantitative adhesion tests have been developed to monitor the mechanical durability of coatings and their adhesion to implants. Since cell adhesion and the resulting osseointegration are significantly influenced by surface wettability, CA measurements are routinely performed to determine the degree of hydrophilicity/hydrophobicity of various surfaces. Surface analysis of coated orthopedic implants is essential in order to understand the final composition of the coatings and their influence on simulated biological processes. Future opportunities lie in the development of coatings that simultaneously promote osseointegration and allow personalized multi-drug delivery, in which the release kinetics, drug type, drug concentration, and consequently therapeutic efficacy are tailored to the needs of the individual. The constantly evolving state-of-the-art characterization techniques will undoubtedly eliminate the current limitations and enable the optimized development of high-value medical implants.

## Figures and Tables

**Table 1:** A list of the chemical structures of bioactive coatings as a function of wavenumbers (cm^−1^) obtained using FTIR/ATR-FTIR.

Drug/Bioactive Coating Material	Functional Groups	Wavenumber (cm^−1^)	References
**Drugs**			
Clindamycin	N–C=O stretching	1682 and 1550	[32]
	C–O stretching	1038 and 1079	
	S–C–H bending	1209 and 1249	
DCF	R–C=O stretching	1305, 1500–1750	[24,93]
	R=C=O stretching	1577	
	C–Cl stretching	650–780	
	HC–N–CH bending	1376	
	CH_2_ bending	1462	
Vancomycin	C–H	3284	[92]
Rifampicin	C–H	2880	[87,92]
	OH−	3480	
	furanone (C=O)	1644	
	acetyl (C=O)	1725	
	(C=O)	1567	
	N–CH_3_	2878	
Enrofloxacin	C=O	1731	[85]
	OH bending	1631	
	COO–stretching	1508	
	COO^−^	1477	
Dexamethason	P–O	1041	[86]
Ibuprofen	C=O stretching	1720	[89]
	C=C of the phenyl ring	1513	
	C–H	1463 and 1378	
**Polymers**			
PCL	asymmetric C=H stretching	2939	[87,94]
	symmetric C=H stretching	2864	
	C=O stretching	1730	
	C–O/C–C stretching	1294	
	asymmetric C–O–C stretching	1240	
	asymmetric CH_2_ stretching	2944 and 2865	
Poly(lactic-co glycolic acid) (PLGA)	C–H	3000–2850	[92]
CHI	O–H	3700–3000	[34,82,93,95]
	Amide NHCOCH_3_	1643 and 1540	
	NH_2_ stretching	3430	
	NH stretching	1654	
	CH scissoring	1422–1380	
	CH stretching	2920	
	C–O–C stretching	900; 1015;	
Poly(methyl methacrylate) (PMMA)	C–O–C symmetric stretching	1149	[88]
	CH_2_ bending	1439	
	C=O stretching	1721	
	asymmetric CH_3_ stretching	2951	
Polyvinyl alcohol (PVA)	OH stretching	3520	[34]
	OH bending	1440	
	asymmetric CH_2_ stretching	2910	
	C–O stretching	1067	
	C=O stretching	1740	
Alginate	symmetric COO−stretching	1620 and 1413	[96]
	COOH stretching	1723	
Carboxymethyl cellulose (CMC)	Carboxylate group	1630	[32]
	O–H	3200–3400	
	C–OH stretching	1000–1250	[24]
	C–O–C bending		
Polydopamine	OH stretching	3348	[86]
	NH stretching	3176	
**Other bioactive materials**			
HA	PO_4_^3−^	956, 1055, and 1101; 567, 603, and 1032;	[39,67,69]
	CO_2_^3−^	854, 1410	
	OH^–^	3484	
BG	Si–O–Si bending	450	[82,88]
	Si–O–Si stretching	930; 1030;	
	P–O stretching	1015	
TNTs	Ti–O	480; 696	[85,97]
	TiO–H	3396	
	Ti-O-Ti	540	
TiO_2_	Ti–O	800	[88]
Single-walled carbon nanotubes	C–O	1112	[67]
	C–C	1630	
	C=O	1730	
	O–H	3440	

**Table 2:** AFM measurements of bioactive coatings on metallic substrates.

Coating Deposition Technique	Implant and Coating Materials	AFM Specifications	Information Obtained	References
Anodization	Ti substrate, TNT coatings	Tapping mode, tapping cantilever tips (NSC15/NoAl), a scanning size of 1.0 µm^2^	Higher surface roughness compared to bare substrate, which contributes to an increase in the osteoblast adhesion and osseointegration of the implant material	[101]
Layer-by-layer	Ti6Al4V alloy and AISI316LVM substrates, poly(2-hydroxyethyl methacrylate) (PHEMA), poly(2-hydroxypropylmethacrylate) (PHPMA), and sodium deoxycholate (NaDOC) coatings containing the anti-inflammatory drug DCF	Acoustic mode, in air at 25 °C, a scanning size of 1 × 1 µm^2^	The coatings are structured in a particle-like form in the case of a polymer layer in the uppermost position, low values of roughness parameters with a decrease in scan size indicate flat morphologies on the substrate and very smooth coating layers	[57]
	AISI316LVM substrate, CHI and DCF coating layers	Tapping mode at room temperature, a sample size of 5 × 5 μm^2^, a resolution of 2048 × 2048 pixels, silicon cantilevers with a resonance frequency of 210–490 kHz, and a force constant of 12–110 N m^−1^	Smaller and thinner surface interconnects in the case of a polymer layer in the uppermost position	[93]
	AISI316LVM substrate, CMC and DCF coating layers	Acoustic mode with scan sizes of 10 × 10, 5 × 5, and 1 × 1 μm^2^, a resolution of at least 512 × 512 pixels, silicon cantilevers with a resonance frequency of 210–490 kHz and a force constant of 12–110 N m^−1^	All samples showed similar results regarding topography, with substrate lines visible on all samples due to the grinding process, the roughness parameters slightly increased for the samples with the DCF layers on top	[24]
	Ti substrate, coatings of poly(acrylic acid) (PAA) and poly(l-lysine) (PLL), with beta cyclodextrin (β-CD) complexes used to retain tetracycline (TC)	Peak-force tapping mode, silicon nitride cantilevers, a nominal spring constant of 0.7 N/m, and a scanning size of 2 × 2 mm	Significantly decreased values of roughness, suggesting that the incorporation of TC/anionic β-CD macromolecules smoothens the surface	[102]
3D printing, electrospinning	3D printing: Ti6Al4V and AISI 316LVM substrates, coatings of cellulose nanofibril suspension, alginate, and CMC, loaded with clindamycin Electrospinning: TiAlV and AISI 316LVM substrates, coatings of CMC and polyethylene oxide, loaded with clindamycin	Tapping mode, room temperature, silicon cantilevers, a resonance frequency of 210–490 kHz, a force constant of 12–110 N m^−1^, scanning sizes of 10 × 10 and 1 × 1 μm^2^, a resolution of 512 × 512 pixels	Relatively smooth surface of the noncoated coatings, functionalization with clindamycin showed no significant effect on the morphology and roughness of the samples, indicating a homogeneous distribution of the drug in the coating	[32]
Grafting	Ti6Al4V substrate, coatings of polymers bearing sulfonate (styrene sodium sulfonate, NaSS) and carboxylate (methylacrylic acid, MA) groups	Contact mode, NP-S tips, a scan rate of 3.3 Hz, two images per sample were acquired from 41 × 41 µm^2^ areas and flattened by first-order line flattening	Increasing the oxidation treatment time from 1 to 3 min. resulted in a doubling of the surface roughness	[60]
Drop casting	Ti substrate, CH/PVA coatings	Tapping mode, a scanning size of 1 × 1 μm^2^, the images are first-order x–y plane fitted and then first-order flattened using Nanoscope software (v1.30)	Nanometer-sized islands throughout the CH/PVA composite films, roughness, which promotes cell adhesion and proliferation, increased with the coating concentration	[34]
Initiated chemical vapor deposition (iCVD)	Ti substrate, coatings of rhBMP-2 immobilized on glycidyl methacrylate (GMA)	A scanning area of 10 × 10 μm ^2^, room temperature	Smooth, bare, and pGMA-coated surface, a rough surface after functionalization with rhBMP-2	[71]

**Table 3:** Kinetic models and their associated equations [205,207,208].

Kinetic Models	Equations	
Zero-order	$\frac{dc}{dt}=-k$	(4)
	where c represents the concentration, *t* represents time, and k represents the release rate constant.	
First-order	$\frac{dc}{dt}=-k\cdot c$	(5)
Higuchi	$fi=Q=\sqrt{D\cdot \left(2c_{t}-c_{s}\right)\cdot c_{s}\cdot t},$	(6)
	or a simplified Higuchi equation:	
	$c_{t}={\;K}_{H}\cdot \sqrt{t}$	(7)
	where Q represents the amount of released drug at a given time and area, $c_{t}$ represents the concentration at time *t*, *c_s_* represents the drug solubility in the media matrix, D represents the diffusion coefficient, and $K_{H}$ represents the Higuchi release rate constant.	
Hixon–Crowell	$\sqrt[{3}]{c_{0}}-\sqrt[{3}]{c_{t}}=K_{\mathrm{HC}}\cdot t$	(8)
	where $K_{\mathrm{HC}}$ represents the Hixon–Crowell release rate constant.	
Korsmeyer–Peppas	$\frac{C_{t}}{C_{\infty }}=K\cdot t^{n}$	(9)
	where $c_{\infty }$ represents the equilibrium drug concentration, K represents the release rate constant, and n the release exponent.	
Baker–Lonsdale	$\frac{3}{2}{\left(1-\frac{C_{t}}{C_{\infty }}\right)}^{\frac{2}{3}}\frac{C_{t}}{C_{\infty }}=K\cdot t$	(10)

**Table 4:** Coating-implant systems, the incorporated drugs, and the drug release characteristics.

System	Drug	Drug Release	Reference
**Electrospinning**			
PCL/HA nanoparticle composite coatings on AZ31 Mg alloy	Simvastatin	Initial burst release controlled by diffusion (first day), followed by sustained release for up to 6 days controlled by polymer degradation	[94]
PLGA on Ti	Aspirin	Prolonged release: early rapid release (50–60% in the first 2 weeks) followed by a slow release for up to 2 months	[108]
	Vancomycin	Biphasic release pattern: initial burst release on day 1, followed by slow and controlled release for up to 28 days	[193]
PCL/HA on Ti	Rifampicin	Initial burst release (40% in the first day), followed by sustained release for 32 days	[87]
**Electrophoretic deposition**			
CaP on Mg alloy	Zoledronate	Continual gradient increase until 1 week	[30]
CHI/BG on AISI 316LVM	Gentamicin	Initial burst release in the first week, slow release for up to 56 days	[41]
halloysite nanotubes/CHI/BG on AISI 315 LVM	Tetracycline hydrochloride	Rapid release within the first 14 days (54% of the drug), followed by slower release for up to 42 days (73% of the drug)	[114]
**Electrophoretic deposition + sol-gel**			
CHI/mesoporous silica nanoparticles on Ti	Ibuprofen	Plateau within the first day	[89]
**Drop casting**			
CHI/amino-functionalized BG on Ti	Vancomycin	Burst release of 42% in the early stages, slow release for up to 14 days	[20]
**Dip coating**			
HA hydrogel on dental implants (Implantium, Dentum Co. Ltd., Suwon, Korea)	rhBMP2	Slow and sustained release for up to 35 days	[40]
**Sol-gel**			
HA/BMP2 on Ti	Gentamicin	Release of more than 99% of gentamicin contained in the coating after 2 days	[13]
**Biomimetic deposition**			
HA on Ti6Al4V	Tobramycin	Initial rapid release, followed by a plateau, 90% of the drug released within 180 min	[36]
Carbonated HA on Ti	Cephalothin, cefamandole, tobramycin and gentamicin	Rapid release: all of the gentamicin within 1 h, 80–90% of the cefamandole and tobramycin within 8 h, 70% of the cephalothin within 16 h	[210]
**LbL deposition**			
CMC on AISI 316LVM	DCF	1–10 min: burst release following the zero-order release mechanism, 10–30 min: fast release (60% of the drug is released by this point) following the Higuchi release mechanism, 30–360 min: slow release, Higuchi, 360–1440 min: the plateau	[24]
CHI/gelatin on Ti	Levofloxacine	Gradual release for up to 4 days	[106]
PAA/PLL/β-CD	Tetracycline	Burst release within the first day, continuous release over the next 15 days, plateau for up to 30 days	[102]
**Anodization (TNTs)**			
TNT/polydopamine	Dexamethasone	Slow release over a period of 75 h (maximum drug release is 84%)	[86]
Periodically tailored TNTs	Indomethacin	A zero-order release mechanism, slow and steady release for up to 17 days (maximum drug release of 50%)	[213]
(-NH_2_)- and (-SH)-treated TNTs	Enrofloxacin	Higuchi release mechanism, initial burst release for up to 7 h, followed by slower matrix-controlled release for up to 50 h	[85]
1–10 layers of PLGA on TNTs	Ibuprofen	Prolonged and controlled release for up to 40 days with 10 layers of PLGA	[37]
Silk fibroin on TNTs	Vancomycin	Higuchi release mechanism, initial burst release on the first day (20% of drug released), followed by a slower release, but constant release for up to 40 days	[214]
Alendronate-grafted HA on TNTs	Raloxifene	Steady and sustained release until 92 h, release rate gradually decreasing from 92 h to 192 h	[211]
**Co-immobilization**			
Sr-HA nanocrystals co-immobilized on AZ31 Mg alloy with polydopamine and carboxymethyl CHI	Alendronate	Initial fast release of 25% of the drug within the first day, followed by slower release with 1–4% of the drug released per day for up to 14 days	[215]

## Data Availability

The raw/processed data required to reproduce these findings cannot be shared at this time due to legal or ethical reasons.

## Abbreviations

AC	alternating current
AFM	atomic force microscopy
ANAB	accelerated neutral atom beam
ASTM	American Society for Testing and Materials
ATR-FTIR	attenuated total reflection Fourier transform infrared spectroscopy
BE	binding energy
BG	bioactive glass
CA	contact angle
CaP	calcium phosphate
CHI	chitosan
CMC	carboxymethyl cellulose
CT	computed tomography
DC	direct current
DCF	diclofenac
EDX/EDXS	energy dispersive X-ray spectroscopy
FTIR	Fourier transform infrared spectroscopy
FTT	Fast Fourier Transform
GCIB	gas cluster ion beam
GMA	glycidyl methacrylate
HA	Hydroxyapatite
HAADF	high-angle annular dark field
LbL	layer-by-layer
MAO	microarc oxidation
MCD	microcrystalline diamond
MH	microwave hydrothermal treatment
NCD	nanocrystalline diamond
NSAIDs	nonsteroidal anti-inflammatory drugs
nTiO_2_	nano titania
PAA	poly(acrylic acid)
PCL	poly(ε-caprolactone)
PEEK	polyether ether ketone
PLGA	poly(lactic-co glycolic acid)
PLL	poly(l-lysine)
PVA	polyvinyl alcohol
QCM	quartz crystal microbalance
QCM-D	quartz crystal microbalance with dissipation monitoring
SAXS	small-angle X-ray scattering
SBF	simulated body fluids
SEM	scanning electron microscopy
Sr-HT	strontium-substituted hardystonite
STM	scanning tunnelling microscopy
TEM	transmission electron microscopy
TEOS–MTES	tetraethylorthosilicate–methyltriethoxysilane
TNTs	titania nanotubes
ToF-SIMS	time-on-flight secondary ion mass spectrometry
USP	United States Pharmacopeia
XPS	X-ray photoelectron spectroscopy
β-CD	beta cyclodextrin
